# MUSCLEMOTION: A Versatile Open Software Tool to Quantify Cardiomyocyte and Cardiac Muscle Contraction In Vitro and In Vivo

**DOI:** 10.1161/CIRCRESAHA.117.312067

**Published:** 2018-02-01

**Authors:** Luca Sala, Berend J. van Meer, Leon G.J. Tertoolen, Jeroen Bakkers, Milena Bellin, Richard P. Davis, Chris Denning, Michel A.E. Dieben, Thomas Eschenhagen, Elisa Giacomelli, Catarina Grandela, Arne Hansen, Eduard R. Holman, Monique R.M. Jongbloed, Sarah M. Kamel, Charlotte D. Koopman, Quentin Lachaud, Ingra Mannhardt, Mervyn P.H. Mol, Diogo Mosqueira, Valeria V. Orlova, Robert Passier, Marcelo C. Ribeiro, Umber Saleem, Godfrey L. Smith, Francis L. Burton, Christine L. Mummery

**Affiliations:** From the Department of Anatomy and Embryology, Leiden University Medical Center, The Netherlands (L.S., B.J.v.M., L.G.J.T., M.B., R.P.D., M.A.E.D., E.G., C.G., M.R.M.J., M.P.H.M., V.V.O., R.P., C.L.M.); Institute of Cardiovascular and Medical Sciences, College of Medical, Veterinary, and Life Science, University of Glasgow, United Kingdom (Q.L., G.L.S., F.L.B.); Hubrecht Institute – Royal Netherlands Academy of Arts and Sciences, Utrecht, The Netherlands (J.B., S.M.K., C.D.K.); Department of Stem Cell Biology, University of Nottingham, University Park, Nottingham, United Kingdom (C.D., D.M.); Department of Experimental Pharmacology and Toxicology, University Medical Center Hamburg Eppendorf, Germany (T.E., A.H., I.M., U.S.); DZHK (German Centre for Cardiovascular Research), Partner Site Hamburg/Kiel/Lübeck (T.E., A.H., I.M., U.S.); Department of Experimental Pharmacology and Toxicology, University Medical Center Hamburg Eppendorf, Hamburg, Germany (U.S.); Hart Long Centrum, Leiden University Medical Center, The Netherlands (E.R.H., M.R.M.J.); Department of Applied Stem Cell Technologies, University of Twente, Enschede, The Netherlands (R.P., M.C.R., C.L.M.).; Division of Heart and Lungs, Department of Medical Physiology, University Medical Center Utrecht, The Netherlands (J.B., S.M.K., C.D.K.); and Clyde Biosciences, Ltd, BioCity Scotland, United Kingdom (G.L.S., F.L.B.).

**Keywords:** arrhythmias, cardiac, humans, pluripotent stem cells, software, zebrafish

## Abstract

**Rationale:**

There are several methods to measure cardiomyocyte and muscle contraction, but these require customized hardware, expensive apparatus, and advanced informatics or can only be used in single experimental models. Consequently, data and techniques have been difficult to reproduce across models and laboratories, analysis is time consuming, and only specialist researchers can quantify data.

**Objective:**

Here, we describe and validate an automated, open-source software tool (MUSCLEMOTION) adaptable for use with standard laboratory and clinical imaging equipment that enables quantitative analysis of normal cardiac contraction, disease phenotypes, and pharmacological responses.

**Methods and Results:**

MUSCLEMOTION allowed rapid and easy measurement of movement from high-speed movies in (1) 1-dimensional in vitro models, such as isolated adult and human pluripotent stem cell-derived cardiomyocytes; (2) 2-dimensional in vitro models, such as beating cardiomyocyte monolayers or small clusters of human pluripotent stem cell-derived cardiomyocytes; (3) 3-dimensional multicellular in vitro or in vivo contractile tissues, such as cardiac “organoids,” engineered heart tissues, and zebrafish and human hearts. MUSCLEMOTION was effective under different recording conditions (bright-field microscopy with simultaneous patch-clamp recording, phase contrast microscopy, and traction force microscopy). Outcomes were virtually identical to the current gold standards for contraction measurement, such as optical flow, post deflection, edge-detection systems, or manual analyses. Finally, we used the algorithm to quantify contraction in in vitro and in vivo arrhythmia models and to measure pharmacological responses.

**Conclusions:**

Using a single open-source method for processing video recordings, we obtained reliable pharmacological data and measures of cardiac disease phenotype in experimental cell, animal, and human models.

## Abstract 2

Supplemental Digital Content is available in the text.

The salient feature of cardiomyocytes is their ability to undergo cyclic contraction and relaxation—a feature critical for cardiac function. In many research laboratories and clinical settings it is, therefore, essential that cardiac contraction can be quantified at multiple levels, from single cells to multicellular or intact cardiac tissues. Measurement of contractility is relevant for analysis of disease phenotypes, cardiac safety pharmacology, and longitudinal measures of cardiac function over time, both in vitro and in vivo. In addition, human genotype–phenotype correlations, investigation of cardiac disease mechanisms, and the assessment of cardiotoxicity are increasingly performed on human-induced pluripotent stem cells (hiPSCs) derived from patients.^1–3^ Many of these studies are performed in nonspecialist laboratories, so that it is important that analysis methods are simplified such that they can be used anywhere with access to just standard imaging equipment. Here, we describe a single method with high versatility that can be applied to most imaging outputs of cardiac contraction likely to be encountered in the laboratory or clinic.

**In This Issue, see p 385**

**Meet the First Author, see p 386**

Electric and calcium signals are usually quantified in vitro using established technologies, such as patch-clamp electrophysiology, multielectrode arrays (MEAs), cation-sensitive dyes, or cation-sensitive genetic reporters.^4^ Although experimental details differ among laboratories, the values for these parameters are, with some approximations, comparable across laboratories, cardiomyocyte source, and cell culture configuration (eg, single cells, multicellular 2-dimensional cardiomyocyte monolayers, and 3-dimensional [3D] cultures).^5,6^ However, there is no comparable method for measuring cardiac contraction across multiple platforms, despite this being a crucial functional parameter affected by many diseases or drugs.^7^ We have developed a method to address this that is built on existing algorithms and is fully automated but, most importantly, can be used on videos, image stacks, or image sequences loaded in the open-source image-processing program ImageJ.^8^ Moreover, it is an open-source, dynamic platform that can be expanded, improved, and integrated for customized applications. The method, called MUSCLEMOTION, determines dynamic changes in pixel intensity between image frames and expresses the output as a relative measure of movement during muscle contraction and relaxation. We applied the concept to a range of biomedical and pharmacologically relevant experimental models that included single human pluripotent stem cell-derived cardiomyocytes (hPSC-CMs), patterned or 2-dimensional cultures of hPSC-CMs, cardiac organoids, engineered heart tissues (EHTs), and isolated adult rabbit cardiomyocytes. Results were validated by comparing outputs of the tool with those from 3 established methods for measuring contraction: optical flow, post deflection, and fractional shortening of sarcomere length. These methods have been tailored to (or only work on) specific cell configurations. Traction force microscopy, fractional shortening of sarcomere length, and microposts are predominantly suitable for single cells.^8,9^ Cardiomyocyte edge or perimeter detection is suitable for adult cardiomyocytes but challenging for immature hPSC-CMs because of poorly defined plasma membrane borders and concentric contraction,^10^ whereas large post deflection is suitable for EHTs or small cardiac bundles^11^ but less so for single cells. Our MUSCLEMOTION software by contrast can be used for all of these applications without significant adaptions. Furthermore, it can be used for multiparameter recording conditions and experimental settings using transmitted light microscopy, fluorescent membrane labeling, fluorescent beads embedded in soft substrates, or patch-clamp video recordings. Drug responses to positive and negative inotropic agents were evaluated across 4 different laboratories in multiple cell configurations using MUSCLEMOTION with reliable predictions of drug effects from all laboratories. Furthermore, MUSCLEMOTION was also applicable to optical recordings of zebrafish hearts in vivo, where it represented a significant time saving in analysis and in human echocardiograms. This versatile tool thus provides a rapid and straightforward way to detect disease phenotypes and pharmacological responses in vitro and in vivo.

## Methods

Extended methods are in the Online Data Supplement.

The datasets generated and analyzed during the current study are available from the corresponding authors on reasonable request.

### Code Availability

MUSCLEMOTION source code has been written in the ImageJ Macro Language and is included in the Online Dataset and is available for use and further development.

### Model Cell

**Figure 1.**
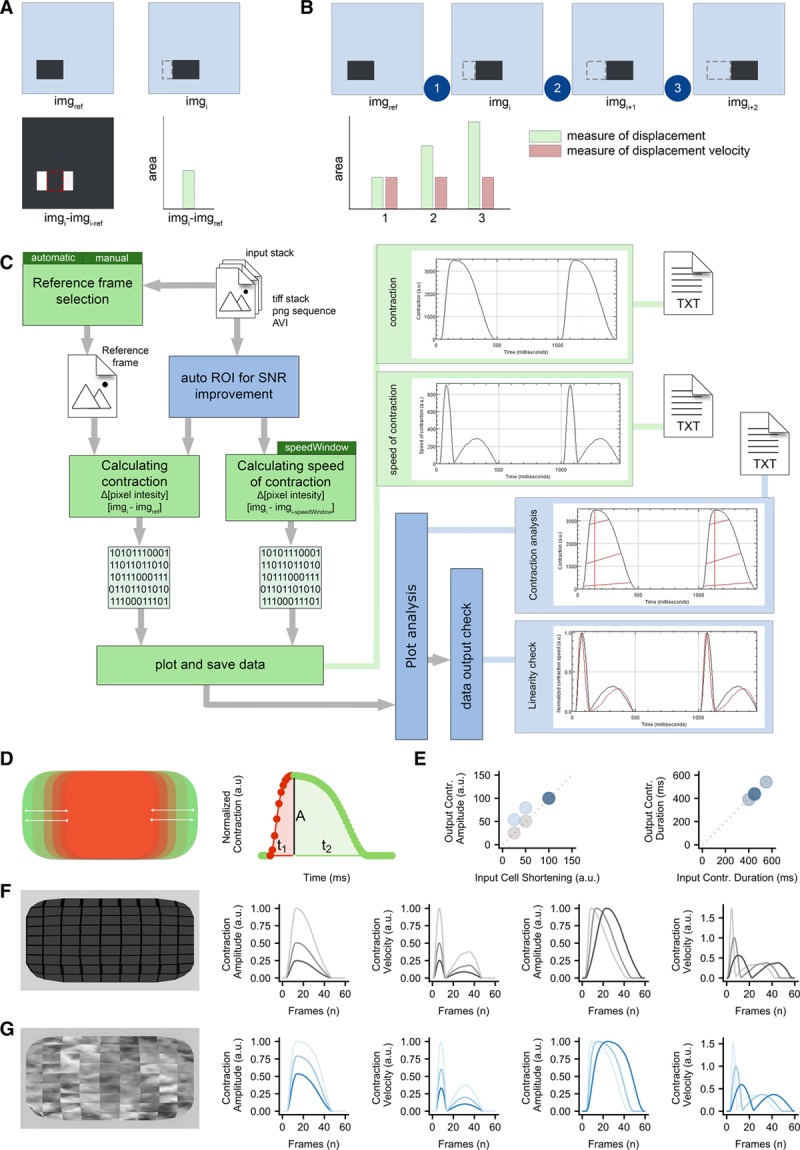
**Algorithm construction and validation.** **A**, Principle of pixel intensity difference by subtraction of *img*_*ref*_ of *img*_*i*_, and measurement of the nonzero area after image subtraction. **B**, Principle of using pixel intensity difference as a measure of displacement and as a measure of displacement velocity. **C**, Schematic overview of MUSCLEMOTION. Green blocks indicate basic steps of the algorithm. Dark green blocks indicate important user input choices. Plots within light green blocks indicate results. Optional steps are shown in blue blocks, with graphical representation of the analyzed parameters indicated by red lines. Three result files are generated containing the raw data: contraction.txt, speed-of-contraction.txt, and overview-results.txt. Furthermore, 3 images showing relevant traces and a log file are generated and saved (not shown in schematic). **D**, Schematic of the contractile pattern of the artificial cell and relative parameters corresponding to amplitude of contraction (**A**), time-to-peak (t_1_), and relaxation time (t_2_). **E**, Correlation between input (*x* axis) and output (*y* axis) parameters used to validate MUSCLEMOTION with 2 artificial cells. **F** and **G**, Frame representing the 2 artificial cells built for MUSCLEMOTION validation and their relative output parameters. SNR indicates signal-to-noise ratio. ROI indicates region of interest.

The in silico cardiomyocyte-like model (Figure 1D, 1F, and 1G) was created using Blender v2.77.

### Optical Flow Analysis

Optical flow analysis was implemented in LabVIEW as described by Hayakawa et al.^12,13^

### Generation of hiPSC-Hypertrophic Cardiomyopathy Isogenic Triplet Using CRISPR/Cas9

Dual guide RNA/Cas9 (clustered regularly interspaced short palindromic repeat–associated 9)-Nickase strategy was designed to introduce the *MYH7* (myosin heavy chain, isoform 7)-C9123T SNP (single-nucleotide polymorphism; encoding the R453C-β-MHC [myosin heavy chain] modification) in ReBl-PAT hiPSC line, as described previously.^14^

### hPSC Culture and Differentiation

hPSCs from multiple independent cell lines (Online Table I) were differentiated to cardiomyocytes as described previously^15–18^ or with the Pluricyte Cardiomyocyte Differentiation Kit (Pluriomics B.V.) according to the manufacturer’s protocol. Experiments were performed at 18 to 30 days after initiation of differentiation, depending on the cell source and configuration. Pluricytes were kindly provided by Pluriomics B.V.

### Patch-Clamp Recordings on hPSC-CMs

Electrophysiological recordings of isolated hPSC-CMs were performed as described previously.^17^

### MEA Recordings of hPSC-CMs

Field potentials from MEAs were recorded and analyzed as published previously.^19^

### Movement of Embedded Beads

Gelatin-patterned polyacrylamide gels containing fluorescent beads were generated and analyzed as described previously.^20^

### Monolayers of hPSC-CMs

Twenty-five thousand to 40 thousand cells were plated per Matrigel-coated glass ø10 mm coverslip.

### Cardiac Organoids

Cardiac organoids composed of hPSC-CMs and hPSC-derived endothelial cells were generated as described previously.^18^

### Adult Cardiomyocytes

Cardiomyocytes were isolated from New Zealand white male rabbits as described previously.^21^

### Membrane Labeling

hPSC-CMs were plated on Matrigel-coated glass-bottom 24-well plates and labeled with CellMask Deep Red according to the manufacturer’s instructions.

### Engineered Heart Tissues

EHTs were generated and analyzed as described previously.^15^

### Zebrafish Hearts

Zebrafish hearts were recorded, treated, and analyzed as described previously.^22^

### Echocardiograms

Anonymized ultrasounds of 5 adult patients were selected from the echocardiography database of the Leiden University Medical Center.

### Statistics

One-way ANOVA for paired or unpaired measurements was applied to test the differences in means on normalized drug effects. *P* values obtained from 2-tailed pairwise comparisons were corrected for multiple testing using Bonferroni method. Statistical analyses were performed with R v3.3.3. *P* values <0.05 were considered statistically significant and indicated with an asterisk (*). *N* values represent biological repeats.

## Results

### Algorithm Development

The principle underlying the algorithm of MUSCLEMOTION is the assessment of contraction using an intuitive approach quantifying absolute changes in pixel intensity between a reference frame and the frame of interest, which can be described as


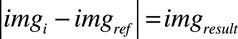


where *img*_*i*_ is the frame of interest, *img*_*ref*_ is the reference frame, and *img*_*result*_ is the resulting image. For every pixel in the frame, each reference pixel is subtracted from the corresponding pixel of interest, and the difference is presented in absolute numbers. Unchanged pixels result in low (black) values, whereas pixels that are highly changed result in high (white) values (Figure 1A). Next, the mean pixel intensity of the resulting image is measured. This is a quantitative measure of how much the pixels have moved compared with the reference frame: more white pixels indicate more changing pixels and, thus, more displacement. When a series of images is analyzed relative to the same reference image, the output describes the accumulated displacement over time (measure of displacement; Figure 1B).

However, if a series of images is analyzed with a reference frame that depends on the frame of interest (eg, *img*_*ref*_ = *img*_*i−1*_), this results in a measure of the relative displacement per interframe interval. We defined this parameter as contraction velocity (measure of velocity; Figure 1B).

Because velocity is the first derivative of displacement in time, the first derivative of the measure of displacement should resemble the measure of velocity derived from image calculations. To test the linearity of the method, 3 movies of moving blocks were analyzed. The block moved back and forth at 2 different speeds in each direction (where 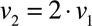): (1) along the *x* axis, (2) along the *y* axis, and (3) along both axes (Online Movie I). As expected, the measure of displacement and velocity showed a linear correlation (Online Figure I). This does not hold when the position of the block in *img*_*i*_ does not overlap the position of the block in *img*_*ref*_, with a consequent saturation in the measure of displacement (ie, max pixel white value; Online Figure II). Therefore, comparison of the differentially derived velocities should approximately overlap in the absence of pixel saturation. This was used as a qualitative parameter to determine whether the algorithm outputs were reliable.

### Algorithm Implementation

MUSCLEMOTION was then modified to handle typical experimental recordings by (1) improving the signal-to-noise ratio, (2) automating reference frame selection, and (3) programming built-in checks to validate the generated output data (Figure 1C). The signal-to-noise ratio was increased by isolating the pixels of interest in a 3-step process: (1) maximum projection of pixel intensity in the complete contraction stack, (2) creation of a binary image of this maximum projection with a threshold level equal to the mean grey value plus SD, and (3) multiplication of the pixel values in this image by the original contraction and speed of the contraction image stack (Online Figure III). This process allowed the algorithm to work on a region of interest with movement above the noise level only.

Next, a method was developed to identify the correct *img*_*ref*_ from the speed of contraction image stack by comparing values obtained from the frame-to-frame calculation with their direct neighboring values, while also checking for the lowest absolute value (Online Figure IV).

The reliability of MUSCLEMOTION for structures with complex movements was validated using a custom-made contracting 3D “synthetic cardiomyocyte” model (Figure 1D, 1F, and 1G) that was adapted to produce contractions with known amplitude and duration. Linearity was preserved during the analysis of the contraction and velocity; other output parameters of the analysis matched the input parameters (Figure 1E). A second 3D model (Figure 1G), with a repetitive pattern aimed to create out-of-bounds problems, was also generated. As expected, contraction amplitude information here was not linear (Figure 1E), although contraction velocity and temporal parameters did remain linear (Figure 1E and 1G). To mitigate this problem, we implemented an option for a 10σ Gaussian blur filter that can be applied on demand to biological samples that presented highly repetitive patterns (eg, sarcomeres in adult cardiomyocytes).

### Algorithm Application to Multiple Cell Configurations and Correlation With Existing Gold Standards

This set of experiments aimed to investigate the versatility of MUSCLEMOTION and examine how its performance compared with standard measures used in each system: (1) optical flow for isolated hPSC-CMs, monolayers, and organoids; (2) post deflection for EHT; and (3) sarcomere length fractional shortening for adult cardiomyocytes. Remarkably, standard methods currently used measure only contraction or contraction velocity. Linearity was preserved in all cases during the analyses, demonstrating the reliability of the results (Online Figure V).

**Figure 2.**
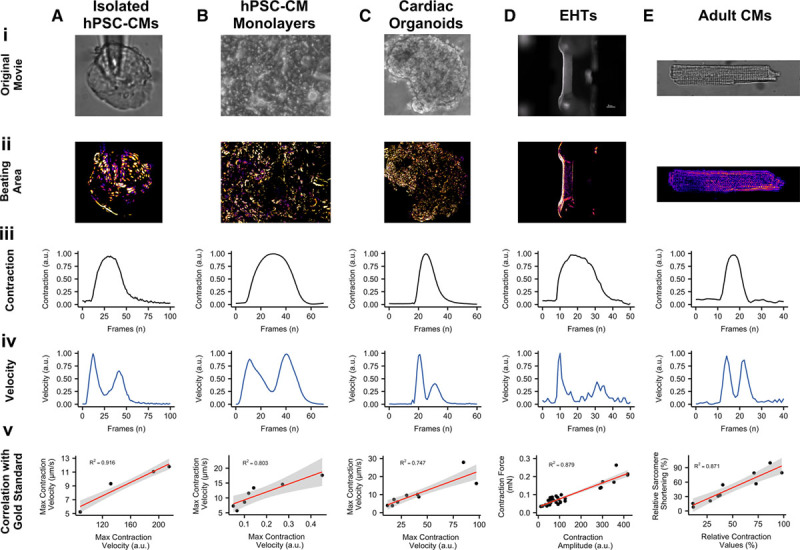
**Correlation of results with gold standards.** **A**, Bright-field image of isolated human pluripotent stem cell-derived cardiomyocytes (hPSC-CMs; **Ai**), with maximum projection step visually enhanced with a fire Look Up Table (**Aii**); contraction (**Aiii**) and velocity (**Aiv**) profiles of each individual beat have been generated by MUSCLEMOTION and temporally aligned; linear regression analysis between MUSCLEMOTION results (*x* axis) and optical flow results (*y* axis; **Av**). **B**, Phase contrast image of hPSC-CM monolayers (**Bi**), with maximum projection step visually enhanced with a fire Look Up Table (**Bii**); contraction (**Biii**) and velocity (**Biv**) profiles of each individual beat have been generated by MUSCLEMOTION and temporally aligned; linear regression analysis between MUSCLEMOTION results (*x* axis) and those obtained with optical flow results (*y* axis; **Bv**). **C**, Phase contrast image of cardiac organoids (**Ci**), with maximum projection step visually enhanced with a fire Look Up Table (**Cii**); contraction (**Ciii**) and velocity (**Civ**) profiles of each individual beat have been generated by MUSCLEMOTION and temporally aligned; linear regression analysis between MUSCLEMOTION results (*x* axis) and those obtained with optical flow results (*y* axis; **Cv**). **D**, Live view of an engineered heart tissue (EHT) during contraction analysis. Scale bar, 1 mm. **Di**, with maximum projection step visually enhanced with a fire Look Up Table (**Dii**); contraction (**Diii**) and velocity (**Div**) profiles of each individual beat have been generated by MUSCLEMOTION and temporally aligned; linear regression analysis between MUSCLEMOTION results (*x* axis) and those obtained with post deflection (*y* axis; **Dv**). **E**, Bright-field image of adult rabbit cardiomyocytes (**Ei**), with maximum projection step visually enhanced with a fire Look Up Table (**Eii**); contraction (**Eiii**) and velocity (**Eiv**) profiles of each individual beat have been generated by MUSCLEMOTION and temporally aligned; linear regression analysis between MUSCLEMOTION results (*x* axis) and those obtained from sarcomere fractional shortening calculation with Fast Fourier Transform (*y* axis; **Ev**). For details on cell sources and cell lines, please refer to Online Table I.

First, single hPSC-CMs (Figure 2A; Online Movie II) exhibited concentric contraction (Figure 2Aii), and contraction velocity amplitudes correlated well with the amplitudes obtained by optical flow analysis (*R*^2^=0.916; Figure 2Av). In contrast to single cells, the area of contraction for hPSC-CM monolayers was distributed heterogeneously throughout the whole field (Figure 2Bii; Online Movie III). Optical flow analysis was compared with our measure of velocity (Figure 2Biv); this showed a good linear correlation (*R*^2^=0.803; Figure 2Bv). Complex (mixed and multicellular) 3D configurations were also investigated by analyzing hPSC-derived cardiac organoids^18^ (Online Movie IV) and EHTs^15^ (Online Movie V). Cardiac organoids showed moderate levels of contraction throughout the tissue (Figure 2Cii), whereas the EHTs showed high deflection throughout the bundle (Figure 2Dii). The contraction velocity of the organoids correlated well with the output of optical flow analysis (*R*^2^=0.747; Figure 2Cv). Similarly, contraction amplitudes in EHTs showed high linear correlation (*R*^2^=0.879) with the absolute force values derived from measurement of pole deflection (Figure 2Dv). Finally, single adult rabbit ventricular cardiomyocytes were analyzed (Figure 2E; Online Movie VI). Large movement was evident around the long edges of the cardiomyocyte (Figure 2Eii). These cells were analyzed with a 10σ Gaussian blur filter, which also minimized (unwanted) effects of transverse movements on contraction patterns. Linearity was preserved (Online Figure V), despite the repetitive pattern of the sarcomeres, and this resulted in accurate measures of both contraction (Figure 2Eiii) and speed of contraction (Figure 2Eiv). The contraction amplitude of the adult cardiomyocytes stimulated at 1 Hz correlated well with the output of sarcomeric shortening using fast Fourier transform analysis^23^ (*R*^2^=0.871; Figure 2Ev). Thus, the MUSCLEMOTION algorithm yielded data in these initial studies comparable with methods of analysis tailored for the individual platforms.

### Application of MUSCLEMOTION to Multiple Imaging and Recording Platforms

**Figure 3.**
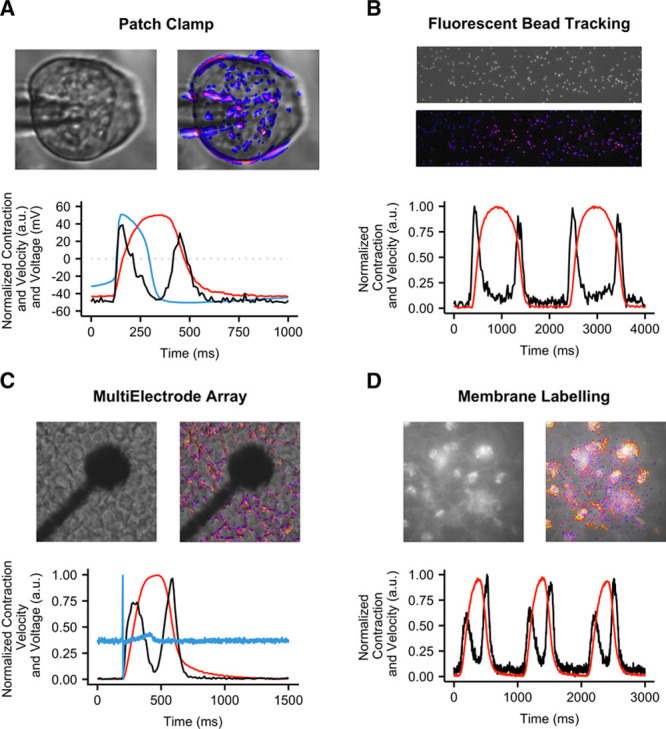
**Application of MUSCLEMOTION to multiple biological situations.** Representative examples with enhancement of moving pixels (**top**) and profiles (**bottom**) of contraction (**A** through **D**, red), velocity (**A** through **D**, black), and voltage (**A** and **C**, blue), respectively, obtained from high-speed movies of patched human pluripotent stem cell-derived cardiomyocytes (hPSC-CMs; **A**), aligned hPSC-CMs on polyacrylamide gels with fluorescent beads (**B**), monolayers of human-induced pluripotent stem cell CMs seeded on multielectrode arrays (**C**), and hPSC-CMs whose membranes have been labeled with CellMask Deep Red (**D**). For details on cell sources and cell lines, please refer to Online Table I.

To examine whether MUSCLEMOTION could potentially be used in applications that measure other aspects of cardiomyocyte functionality in parallel, we first determined the electrophysiological properties of hPSC-CMs using patch clamp while recording their contractile properties through video imaging. This allowed simultaneous quantitative measurement of action potentials and contraction (Figure 3A), for in-depth investigation of their interdependence. We observed a typical^24^ profile of action potential followed by its delayed contraction.

To measure contractile force in combination with contractile velocity in single cardiomyocytes, we integrated fluorescent beads into polyacrylamide substrates patterned with gelatin (Figure 3B), where the displacement of the beads is a measure of cardiomyocyte contractile force^20^ (Online Movie VII). Additionally, field potentials and contraction profiles of hPSC-CMs were analyzed from simultaneous electric and video recordings of monolayers plated on MEAs (Figure 3C; Online Movie VIII).

Similarly, effective quantification of contraction profiles was obtained for fluorescently labeled hPSC-CM monolayer cultures (Figure 3D; Online Movie IX), allowing MUSCLEMOTION to be integrated on high-speed fluorescent microscope systems for automated data analysis.

### Application of MUSCLEMOTION to Drug Responses in Different Cell Models in Different Laboratories

Having shown that MUSCLEMOTION was fit-for-purpose in analyzing contraction over a variety of platforms, we next sought to demonstrate its ability to detect the effects of positive and negative inotropes. This is essential for ensuring the scalability of the tool over multiple platforms, particularly in the context of hiPSC-CMs where regulatory authorities and pharmaceutical companies are interested in using these cells as human heart models for drug discovery, target validation, or safety pharmacology.^25^ For isoprenaline and nifedipine, the main parameters of interest are contraction amplitude (isoprenaline and nifedipine), relaxation time (isoprenaline), and contraction duration (nifedipine).

**Figure 4.**
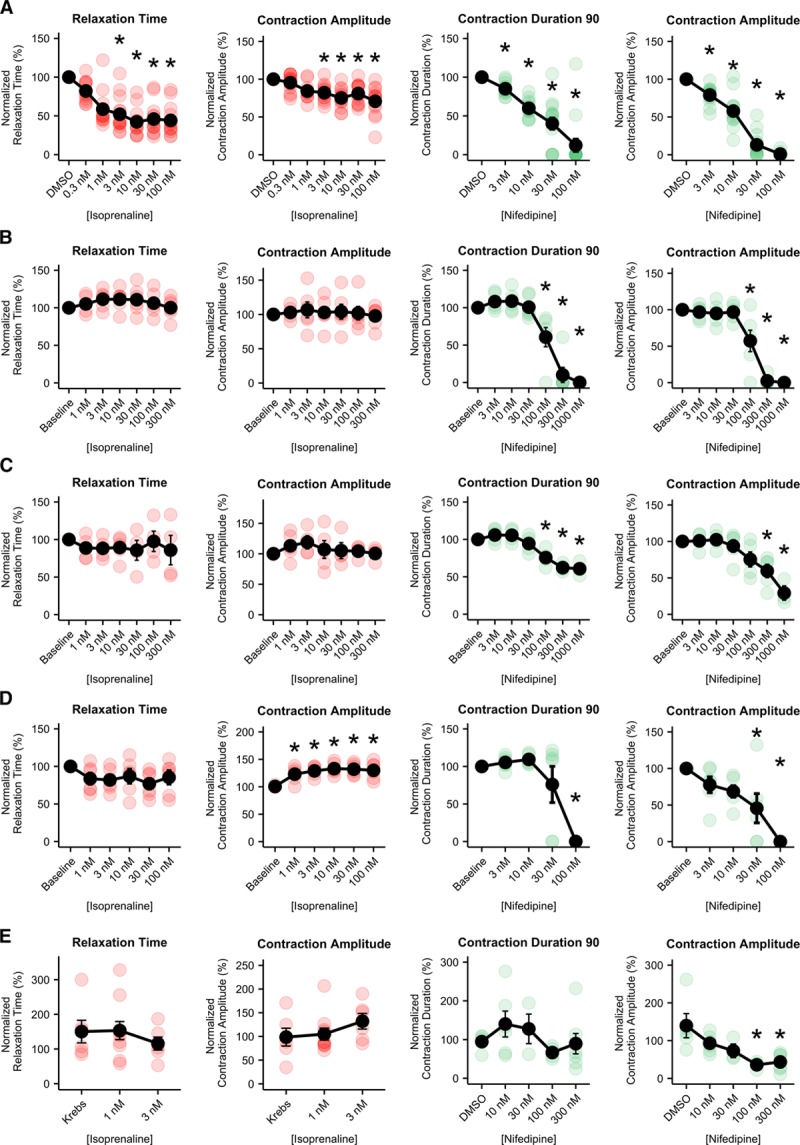
**Pharmacological challenge with positive and negative inotropic compounds.** **A**, Average dose–response curves (black traces) and single measurements for several parameters obtained in isolated, spontaneously beating, aligned human pluripotent stem cell-derived cardiomyocytes (hPSC-CMs) treated with isoprenaline (**left**, red) and nifedipine (**right**, green). **B**, Average dose–response curves (black traces) and single measurements for several parameters obtained from monolayers of hPSC-CMs treated with isoprenaline (**left**, red) and nifedipine (**right**, green). **C**, Average dose–response curves (black traces) and single measurements for several parameters obtained in cardiac organoids treated with isoprenaline (**left**, red) and nifedipine (**right**, green). **D**, Average dose–response curves (black traces) and single measurements for several parameters obtained in engineered heart tissues treated with isoprenaline (**left**, red) and nifedipine (**right**, green). **E**, Average dose–response curves (black traces) and single measurements for several parameters obtained in adult rabbit cardiomyocytes treated with isoprenaline (**left**, red) and verapamil (**right**, green). Average data points (black) represent mean±standard error of the mean. For details on cell sources and cell lines, please refer to Online Table I. Data information: *P* values DMSO (dimethyl sulfoxide) vs dose. **Ai**, 0.3 nmol/L, 0.2897; 1 nmol/L, 3.4/10^6^; 3 nmol/L, 3.8/10^8^; 10 nmol/L, 7/10^11^; 30 nmol/L, 7.3/10^10^; and 100 nmol/L, 2.4/10^10^. **Aii**, 0.3 nmol/L, 1; 1 nmol/L, 0.0645; 3 nmol/L, 0.0136; 10 nmol/L, 8.2/10^5^; 30 nmol/L, 0.0063; and 100 nmol/L, 2.4/10^6^. *N*=14; 14; 14; 14; 14; 14; 14. **Aiii**, 3 nmol/L, 0.6533; 10 nmol/L, 4/10^5^; 30 nmol/L, 2/10^9^; and 100 nmol/L, 1.5/10^15^. **Aiv**, 3 nmol/L, 0.00 054; 10 nmol/L, 1.9/10^11^; 30 nmol/L, <2/10^16^; and 100 nmol/L, <2/10^16^. *N*=14; 14; 14; 14; 14. *P* values baseline vs dose. **Bi**, 1 nmol/L, 1; 3 nmol/L, 1; 10 nmol/L, 1; 30 nmol/L, 1; 100 nmol/L, 1; and 300 nmol/L, 1. **Bii**, 1 nmol/L, 1; 3 nmol/L, 1; 10 nmol/L, 1; 30 nmol/L, 1; 100 nmol/L, 1; and 300 nmol/L, 1. *N*=6; 5; 6; 6; 6; 6; 6. **Biii**, 3 nmol/L, 1; 10 nmol/L, 1; 30 nmol/L, 1; 100 nmol/L, 0.00 801; 300 nmol/L, 2.7/10^9^; and 1000 nmol/L, 1.8/10^10^. **Biv**, 3 nmol/L, 1; 10 nmol/L, 1; 30 nmol/L, 1; 100 nmol/L, 0.00 084; 300 nmol/L, 2.9/10^11^; and 1000 nmol/L, 1.5/10^11^. *N*=6; 6; 6; 6; 6; 6; 6. *P* values baseline vs dose. **Ci**, 1 nmol/L, 1; 3 nmol/L, 1; 10 nmol/L, 1; 30 nmol/L, 1; 100 nmol/L, 1; and 300 nmol/L, 1. **Cii**, 1 nmol/L, 1; 3 nmol/L, 1; 10 nmol/L, 1; 30 nmol/L, 1; 100 nmol/L, 1; and 300 nmol/L, 1. *N*=5; 5; 4; 5; 4; 4; 4. **Ciii**, 3 nmol/L, 1; 10 nmol/L, 1; 30 nmol/L, 1; 100 nmol/L, 0.00 181; 300 nmol/L, 2.9/10^6^; and 1000 nmol/L, 1.7/10^5^. **Civ**, 3 nmol/L, 1; 10 nmol/L, 1; 30 nmol/L, 1; 100 nmol/L, 0.54 836; 300 nmol/L, 0.01 392; and 1000 nmol/L, 8.2/10^5^. *N*=5; 5; 4; 5; 5; 5; 3. *P* values baseline vs dose. **Di**, 1 nmol/L, 1; 3 nmol/L, 1; 10 nmol/L, 1; 30 nmol/L, 0.47; and 100 nmol/L, 1. **Dii**, 1 nmol/L, 0.02 318; 3 nmol/L, 0.00 170; 10 nmol/L, 0.00 028; 30 nmol/L, 0.00 044; and 100 nmol/L, 0.00 113. *N*=5; 5; 5; 5; 5; 5. **Diii**, 3 nmol/L, 1; 10 nmol/L, 1; 30 nmol/L, 1; and 100 nmol/L, 3/10^5^. **Div**, 3 nmol/L, 1; 10 nmol/L, 0.49 856; 30 nmol/L, 0.01 473; and 100 nmol/L, 7/10^6^. *N*=6; 6; 6; 6; 6. *P* values Krebs vs dose. **Ei**, 1 nmol/L, 1; 3 nmol/L, 1. **Eii**, 1 nmol/L, 1; and 3 nmol/L, 0.54. *N*=6; 10; 7. *P* values DMSO vs dose. **Eiii**, 10 nmol/L, 1; 30 nmol/L, 1; 100 nmol/L, 1; and 300 nmol/L, 1. **Eiv**, 10 nmol/L, 0.5298; 30 nmol/L, 0.2470; 100 nmol/L, 0.0054; and 300 nmol/L, 0.0029. *N*=7; 8; 4; 5; 7. *P<0.05

The relaxation time of spontaneously beating isolated hPSC-CMs on gelatin-patterned polyacrylamide substrates treated with isoprenaline significantly decreased as expected at doses >1 nmol/L. Similar to what has been reported,^26^ contraction amplitude decreased at doses >1 nmol/L. Nifedipine treatment decreased both contraction amplitude and duration starting from 3 nmol/L, respectively (Figure 4A). In paced (1.5 Hz) hPSC-CM monolayers, no significant effects were measured after addition of isoprenaline on either relaxation time or contraction amplitude. Nifedipine caused a progressive decrease in contraction duration and amplitude in a concentration-dependent manner starting at 100 nmol/L (Figure 4B). Similarly, cardiac organoids paced at 1.5 Hz showed no significant effects on both relaxation time and contraction amplitude with isoprenaline, whereas both parameters decreased after nifedipine, starting from 100 and 300 nmol/L, respectively (Figure 4C). EHTs paced at 1.5× baseline frequency and analyzed with MUSCLEMOTION showed a positive inotropic effect starting from 1 nmol/L isoprenaline and a negative inotropic effect starting at 30 nmol/L nifedipine as reported previously^15^ (Figure 4D).

Paced (1 Hz) adult rabbit cardiomyocytes exhibited no significant increase in relaxation time and contraction amplitude at any isoprenaline concentration. At concentrations >3 nmol/L, adult cardiomyocytes exhibited after-contractions and triggered activity during diastole, which hampered their ability to be paced at a fixed frequency. No significant effects were observed on contraction duration with nifedipine, whereas contraction amplitude significantly decreased in a dose-dependent manner starting from 100 nmol/L (Figure 4E). Data generated by post deflection and sarcomere fractional shortening are available for comparison purposes in Online Figure VI.

### Analysis of Disease Phenotypes In Vitro

**Figure 5.**
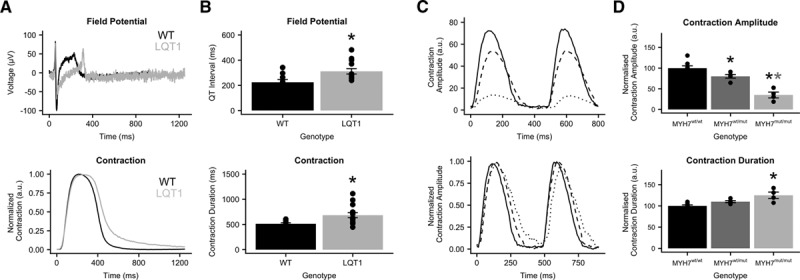
**In vitro disease phenotypes.** **A**, Field potential and contraction profile of wild-type (black) and long-QT syndrome type 1 (LQT1; gray) human pluripotent stem cell-derived cardiomyocyte monolayers on multielectrode arrays. **B**, Quantitative analysis of the QT interval and the contraction duration. **C**, Raw (**top**) and normalized (**bottom**) contraction profiles of hypertrophic cardiomyopathy–engineered heart tissues from MYH7 (myosin heavy chain, isoform 7)^wt/wt^ (solid), MYH7^wt/mut^ (dashed), and MYH7^mut/mut^ (dotted) cell lines. **D**, Quantitative analysis of contraction amplitude and contraction duration. Data information: *P* values QT interval: wild type (WT) vs LQT1, 0.012. *P* values contraction duration: WT vs LQT1, 0.012. *P* values contraction amplitude: MYH7^wt/wt^ vs MYH7^wt/mut^, 0.026; MYH7^wt/wt^ vs MYH7^mut/mut^, 6/10^6^; and MYH7^wt/mut^ vs MYH7^mut/mut^, 0.00 065. *P* values contraction duration: MYH7^wt/wt^ vs MYH7^wt/mut^, 0.062; MYH7^wt/wt^ vs MYH7^mut/mut^, 0.00 085; and MYH7^wt/mut^ vs MYH7^mut/mut^, 0.0046. *P<0.05

Contractility of hiPSC-CMs carrying mutations associated with long-QT syndrome type 1^27^ and hypertrophic cardiomyopathy were characterized in distinct cell configurations: monolayers plated on MEAs and EHTs, respectively. As demonstrated previously, long-QT syndrome type 1 phenotype was captured as a prolongation of the QT interval of the field potential.^17,27^ As expected, contraction duration measured with MUSCLEMOTION was also prolonged (Figure 5A and 5B). EHTs were fabricated from an isogenic triplet carrying the MYH7^R453C^ mutation either in homozygosity or heterozygosity and showed a gene dosage effect on the contractility recapitulating disease severity.

### Analysis of Disease Phenotypes In Vivo

**Figure 6.**
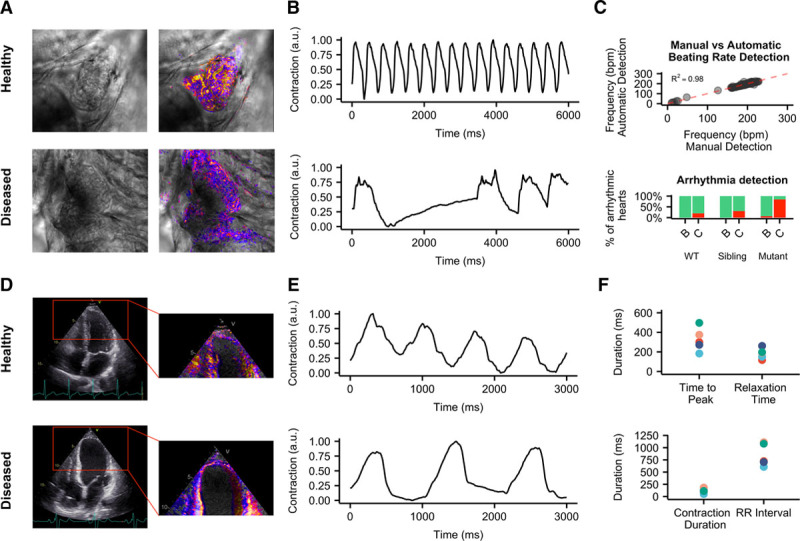
**In vivo disease phenotypes.** **A**, Representative examples of wild-type (**top**) and *gnb5a*/*gnb5b* mutant (**bottom**) zebrafish and relative enhancement of moving pixels. **B**, Representative qualitative analyses of normal (**top**) and arrhythmic (**bottom**) contraction profiles from wild-type and *gnb5a*/*gnb5b* mutant zebrafish treated with carbachol. **C**, Correlation of results obtained from manual (*x* axis) vs automatic (*y* axis) detection of beating frequency (**top**); distribution of normal (green) and arrhythmic (red) contraction patterns in baseline condition (**B**) and after treatment with carbachol (**C**) in wild-type and *gnb5a*/*gnb5b* mutant zebrafish (**bottom**). **D**, Representative echocardiograms of healthy (**top**) and cardiomyopathic (**bottom**) human individuals. Ventricles have been manually cropped, and the enhancement of moving pixels is overlaid. **E**, Representative qualitative analyses of normal (**top**) and poor (**bottom**) ventricular functions. **F**, Quantitative data collected from echocardiogram in 5 individuals. Each color represents 1 individual.

To extend analysis to hearts in vivo, we took advantage of the transparency of zebrafish, which allows recording of contracting cardiac tissue in vivo (Figure 6A; Online Movie X). It was previously shown that mutations in *GNB5* are associated with a multisystem syndrome in human, with severe bradycardia at rest. Zebrafish with loss-of-function mutations in *gnb5a* and *gnb5b* were generated. Consistent with the syndrome manifestation in patients, zebrafish *gnb5a*/*gnb5b* double mutant embryos showed severe bradycardia in response to parasympathetic activation.^22^ Irregularities in heart rate were visually evident and were clearly distinguishable from the wild-type counterpart after analysis with MUSCLEMOTION (Figure 6B). Quantification of the heart rate of these zebrafishes with MUSCLEMOTION highly correlated (*R*^2^=0.98) with the results of the published manual analyses^22^ (Figure 6C). There was, however, a striking time saving for operators in performing the analysis using the algorithm (5–10× faster than manual analysis; 150 recordings were analyzed in 5 hours versus 4 days) without compromising the accuracy of the outcome. Qualitative analysis of contraction patterns allowed rapid discrimination between arrhythmic versus nonarrhythmic responses to carbachol treatment (Figure 6C).

Finally, we examined human echocardiograms from 5 healthy and cardiomyopathic individuals (Figure 6D; Online Movie XI). To assess ventricular function, videos were cropped to exclude movement contributions of the atria and valves. MUSCLEMOTION enabled rapid quantification of temporal parameters from standard ultrasound echography (Figure 6E), such as time-to-peak, relaxation time, R-R interval, and the contraction duration (Figure 6F).

## Discussion

A reliable and easy-to-use method to quantify cardiac muscle contraction would be of significant benefit to many basic and clinical science laboratories to characterize cardiac disease phenotypes, understand underlying disease mechanisms, and predict cardiotoxic effects of drugs.^15,26^ Quantification of frame-to-frame differences in pixel intensity has been used in recent reports with success^10^; however, the full spectrum of applications for which these algorithms are relevant, how their output data correlate with gold standards in each system, and software performance, specifications, license, and software availability, have remained unclear.

Here, we developed and tested a user-friendly, inexpensive, open-source software platform that serves this purpose in a variety of biological systems of heart tissue. Its integration into current research practices would benefit data sharing, reproducibility, comparison, and translation in many clinically relevant contexts.^28^

The linearity and reliability of MUSCLEMOTION were validated using a 3D reconstructed artificial cardiomyocyte, which gave the expected linear correlations between known inputs and the outputs (Figure 1D through 1F). When random repetitive patterns were applied, amplitude outputs differed from inputs, suggesting a potential limitation to measuring contraction amplitudes in highly repetitive biological samples (such as when sarcomere patterns are well organized), whereas temporal parameters remained valid (Figure 1D, 1E, and 1G). However, conditions such as these would be unlikely in standard biological samples, where camera noise significantly reduces the possibility of saturating pixel movement. We partially attenuated this problem by applying, on user demand, a 10σ Gaussian blur filter, which significantly increased the accuracy of MUSCLEMOTION with highly repetitive structures. Also, to increase reliability, we built in additional controls to detect any mismatches and errors. MUSCLEMOTION can automatically identify and select the reference frame and increase the signal-to-noise ratio—features which were particularly relevant in reducing user bias and interaction while improving user experience. MUSCLEMOTION is valid in a wide range of illumination conditions without changing temporal parameters; however, exposure time was linearly correlated with contraction amplitude (Online Figure VII). Furthermore, a series of conditions in which there is no contraction has been used as a negative control (Online Figure VIII). Batch mode analyses and data storage in custom folders were also incorporated to support overnight automated analyses. For accurate quantification of amplitude, time-to-peak, and relaxation time, an appropriate sampling rate should be chosen. For applications similar to those described here, we recommend recording rates >70 frames per second to sample correctly the fast upstroke of the time-to-peak typical of cardiac tissue. This recording rate is easily achievable even using smartphone slow-motion video options (≈120/240 frames per second), obviating the need for dedicated cameras and recording equipment if necessary.

We demonstrated excellent linear correlations between our software tool and multiple other standard methods independent of substrate, cell configuration, and technology platform and showed that MUSCLEMOTION is able to capture contraction in a wide range of in vivo and in vitro applications (Figures 2 and 3). Specifically, we identified several advantages compared with optical flow algorithms in terms of speed and the absence of arbitrary binning factors or thresholds, which, when modified, profoundly affect the results. One limitation compared with optical flow or EHT standard algorithm is that the tool lacks qualitative vector orientation, making it more difficult to assess contraction direction. Particularly important was the correlation with force data calculated from the displacement of flexible posts by EHTs. This indicates that when the mechanical properties of substrates are known,^29^ MUSCLEMOTION allows absolute quantification of contractile force. Technical limitations of the EHT recording system allowed us to analyze only movies with JPEG (Joint Photographic Experts Group) compression; this resulted in loss of pixel information that might have negatively influenced the correlation shown. For better and more accurate results on contraction quantification, nonlossy/uncompressed video formats should be used for recordings because individual pixel information is lost on compression and, therefore, not available for analysis by MUSCLEMOTION.

We proposed and validated practical application in pharmacological challenges using multiple biological preparations recorded in different laboratories; this means that immediate use in multiple independent high-throughput drug-screening pipelines is possible without further software development being required, as recently applied for a drug-screening protocol on cardiac organoids from hPSCs.^18^ Intuitively, the possibility of having interassay comparisons will also be of particular relevance where comparisons of contraction data across multiple platforms are required by regulatory agencies or consortia (eg, CiPA [Comprehensive In Vitro Proarrhythmia Assay] and CSAHi [Consortium for Safety Assessment Using Human iPS Cells]).^5,6,25,30^ Moreover, this might offer a quantitative approach to investigate how genetic or acquired diseases of the heart (eg, cardiomyopathies^7^ and long-QT syndrome^31^), heart failure resulting from anticancer treatments,^32,33^ or maturation strategies^20,34,35^ affect cardiac contraction. The possibility of linking in vitro with in vivo assays, with low-cost technologies applicable with existing hardware, certainly represents an advantage as demonstrated by automatic quantification of zebrafish heartbeats and human echocardiograms (Figure 6). Overall, these results clearly demonstrated that contraction profiles could be derived and quantified in a wide variety of commonly used experimental and clinical settings. MUSCLEMOTION might represent a starting point for a swift screening method to provide clinically relevant insights into regions of limited contractility in the hearts of patients. We encourage further development of this open-source platform to fit specific needs; future areas of application could include skeletal or smooth muscle in the same range of formats described here.

MUSCLEMOTION allows the use of a single, transparent method of analysis of cardiac contraction in many modalities for rapid and reliable identification of disease phenotypes, potential cardiotoxic effects in drug-screening pipelines, and translational comparison of contractile behavior.

### Limitations

Saturation of pixel movements may affect contraction amplitudes. However, as demonstrated with the artificial cardiomyocyte, contraction velocity and all temporal parameters remained valid. We also minimized the impact of highly repetitive structures on the output of MUSCLEMOTION by applying a Gaussian filter, which also helped in reducing the impact of transverse movements on contraction profiles. High-frequency contraction might complicate baseline detection, especially if the duration of the contracted state is similar to that of the relaxed (eg, approaching sinusoidal). We have implemented a fast mode option that captures reliable baseline values even at high contraction rates. MUSCLEMOTION does not measure absolute values of cell shortening or force of contraction. However, as demonstrated by correlations with these physical quantities (Figure 2D and 2Ev), specific setups can be calibrated to obtain such readout.

Adult cardiomyocytes contract dominantly along the longitudinal axis. However, hPSC-CMs are highly variable in shape, often showing concentric contractions, so that effects of transverse movement are usually intrinsic to the experimental model and they should be considered in the global contraction analysis. Indirect transverse movements originating from uncontrolled experimental conditions or moving objects other than those of interest (eg, vibrations, sample shift, floating debris, and air bubbles) should be avoided because they might lead to overestimation of the cardiomyocyte contraction.

## Sources of Funding

This work was initiated in the context of the National Centre for the Replacement, Refinement, and Reduction of Animals in Research CRACK IT InPulse project code 35911 to 259146, with support from GlaxoSmithKline. It was supported by the following grants: ERC-AdG (European Research Council - Advanced Grant) STEMCARDIOVASC (C.L. Mummery, B.J. van Meer, E. Giacomelli, M. Bellin, and L.G.J. Tertoolen), ZonMW (ZorgOnderzoek Nederland - Medische wetenschappen) MKMD (Meer Kennis met Minder Dieren) Applications of Innovations 2015 to 2016 (C.L. Mummery, M. Bellin, and L. Sala), BHF (British Heart Foundation) grants SP/15/9/31605 and PG/14/59/31000 and BIRAX 04BX14CDLG grants (C. Denning), ERC-AdG IndivuHeart (T. Eschenhagen), DZHK (German Centre for Cardiovascular Research; T. Eschenhagen, U. Saleem, A. Hansen, and I. Mannhardt), ERC-StG (European Research Council - Starting Grant) StemCardioRisk (R.P. Davis and M.P.H. Mol), and the Netherlands Organisation for Scientific Research grant VIDI-917.15.303 (R.P. Davis and C. Grandela). The Dutch Heart Foundation: CVON (Cardiovasculair Onderzoek Nederland) 2012–10 Predict project (C.D. Koopman), E-Rare–CoHeart project (S.M. Kamel), and CVON–HUSTCARE 2013 to 2018 (C.L. Mummery and L. Sala).

## Disclosures

C.L. Mummery and R. Passier are cofounders of Pluriomics B.V. G.L. Smith and F.L. Burton are cofounders of Clyde Biosciences, Ltd. T. Eschenhagen, A. Hansen, and I. Mannhardt are cofounders of EHT Technologies GmbH. The other authors report no conflicts.

## Supplementary Material

## Novelty and Significance

### What Is Known?

- Quantification of cardiac contraction in vitro and in vivo is essential to characterize effects of drugs and disease on cardiac myocyte and heart function.
- Most current software tools to measure and quantify contraction require tailored hardware, expensive software licences, arbitrary analysis thresholds, or advanced informatics skills not available in many laboratories.
- Validation of data and reproducibility across different laboratories, platforms, or cell configurations is limited because complex data analysis methodologies are not directly comparable.
