# Supplementary material for: MUSCLEMOTION: A Versatile Open Software Tool to Quantify Cardiomyocyte and Cardiac Muscle Contraction In Vitro and In Vivo
Source: Circ Res. 2018 Feb 1;122(3):e5–e16. doi: 10.1161/CIRCRESAHA.117.312067 (PMC5805275; doi:10.1161/CIRCRESAHA.117.312067)
Supplement: Supplementary file 13 [file res-122-0e5-s013.pdf]

MUSCLEMOTION - User Manual

BJ van Meer, MSc and Luca Sala, PhD

Department of Anatomy and Embryology, Leiden University Medical Center, The Netherlands

2017

Contents

1 Before you begin **1**

2 Algorithm explanation **2**

3 Requirements **3**

4 Limitations **3**

5 Installation **5**

6 Running MUSCLEMOTION **6**

7 Outputs **9**

8 Troubleshooting **12**

9 How to cite **12**

10 Contact **12**

1 Before you begin

MUSCLEMOTION is a powerful and versatile ImageJ macro to measure *in vitro* or *in vivo* muscle contraction or motion. Originally designed for quantifying 2D and 3D stem cell derived cardiomyocytes contraction, it proved applicable to many different cell types, systems and experimental setups. Developed in a collaboration between the Leiden University Medical Center and the University of Glasgow by a combination of biologists and engineers, it aims to provide a simple software tool to quantify contraction without the need for specific knowledge of software or expensive equipment. Furthermore, by making MUSCLEMOTION open source, we encourage reproducible and open analysis protocols and tools for open science.

2 Algorithm explanation

This chapter describes the working of MUSCLEMOTION in detail. It can be used to better understand and interpret the output data, select the correct parameters for analysis or as a guide to developers that would like to improve the algorithm, in general or for a specific purposes. Please refer to Sala L. et al., *bioRχiv* (2017), for further information on the algorithm principle and applicability.

Basic principle

The principle underlying the algorithm is the assessment of contraction using an intuitive approach to quantify absolute changes in pixel intensity between a reference frame and the frame of interest, which can be described as

$$| \, img_i - img_{ref} \, | \quad = \quad img_{result} \tag{1}$$

where img_i is the frame of interest, img_{ref} is the reference frame and img_{result} is the resulting image. For every pixel in the frame, each reference pixel is subtracted from the corresponding pixel of interest and the difference is presented in absolute numbers. Unchanged pixels result in low (black) values, while pixels that are highly changed result in high (white) values (see Figure 1). Next, the mean pixel intensity of the complete resulting image is measured. This a quantitative measure of how much the pixel has moved compared to the reference frame: more white pixels indicate more changing pixels and, thus, more movement. When a series of images is analysed relative to the same reference image, the output describes the accumulated change of displacement over time (see Figure 1). However, if a series of images is analysed with a reference frame that depends on the frame of interest (e.g. $img_{ref} = img_{i-1}$), this results in a measure of the relative difference between pixel intensities within a certain period of time, hence the contraction velocity (see Figure 1).

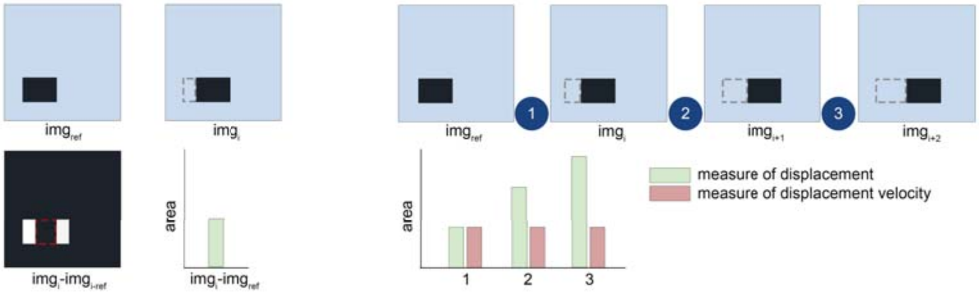

Figure 1: Basic principle underlying MUSCLEMOTION.

An important parameter to understand is the *speedWindow*, which defines the number of frames that are used in the contraction velocity ($img_{ref} = img_{i-speedWindow}$) and

is set at 2 by default; other values can be used an depending on the noise, the frame rate and the contraction duration of the sample that is being analysed. By increasing the *speedWindow*, the noise in the speed of contraction can be reduced but by making the *speedWindow* too large the output might become unreliable because of the high degree of filtering. Whether or not this is the case can be checked by inspection of the output file Comparison calculated (red) and measured (black) speed.jpg (see Figure 10).

3 Requirements

Hardware

MUSCLEMOTION is an image processing tool and heavily relies on RAM usage. However, it is designed to work with file sizes larger than the RAM and is therefore not likely to be limited by the amount of RAM, albeit computational time will be significantly affected. Similar considerations hold for the CPU. A high-speed recording camera, capable of at least 60-75 frames per second, is required for the movie acquisition. A smartphone camera with slow-motion feature (120/240 frames per second) is good as soon as it is steadily mounted on the microscope. Several types of adaptors can be found online.

Software

MUSCLEMOTION runs on any operating system on which ImageJ is operational. MUSCLEMOTION has been developed and tested on ImageJ 1.51n, but is expected to work with every ImageJ version. Please refer to the *Installation* section for more information on how to download and install ImageJ. While MUSCLEMOTION is running, other use of the computer should be avoided.

Input files

MUSCLEMOTION reads four types of files: uncompressed AVI, TIF stacks and PNG or TIF image sequences (ordered alphabetically). Other video formats can be converted with open source video conversion tool like FFmpeg. To ensure correct measurement of the contraction the input files should obey to the Nyquist-Shannon criterium: the recording speed (sample speed) should be at least twice as high as the fastest parameter in the signal. In addition, the sampling speed should not be extreme, as frame to frame differences could then become smaller than the noise. This can be corrected by choosing a higher *speedWindow* (frame-to-frame distance, see Algorithm explanation and advanced options).

4 Limitations

A small number of limitations should be taken in consideration before using MUSCLEMOTION. Since the algorithm is based on changes in pixel intensity, variations of the lighting conditions during measurements should be avoided. Temporal parameters will

remain constant, but the contraction amplitude is changed linearly with an increase in light, as shown in Figure 2.

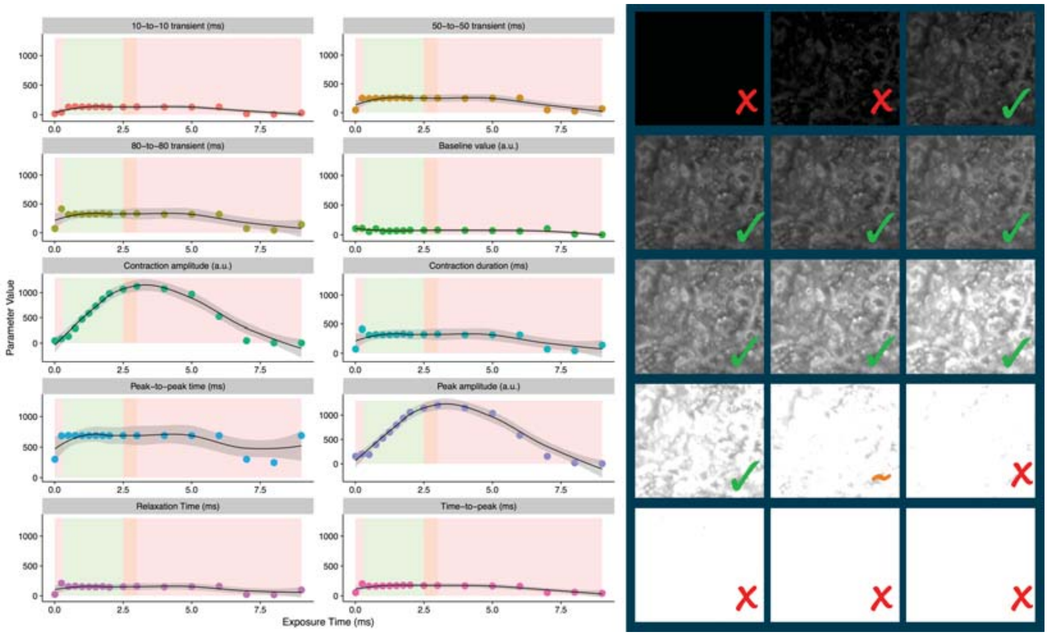

Figure 2: Effect of lighting conditions on output parameters.

If the periodicity of the contraction of interest is very symmetric (i.e. the duration of the contraction is similar in length to the baseline) the correct reference frame might not be detectable by the algorithm. It is therefore advised to check the output carefully, especially at higher contraction frequencies. If a mistaken reference frame has been selected, use the built-in manual reference frame selection tool (Figure 3).

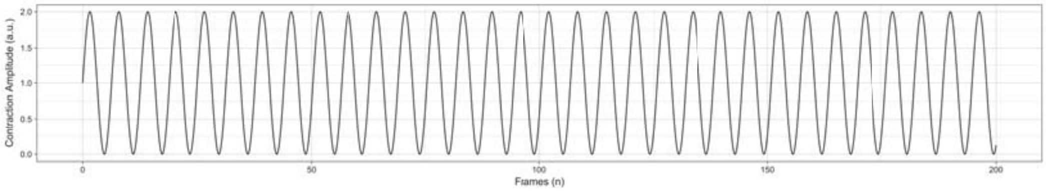

Figure 3: Example of contraction profile that might confound MUSCLEMOTION's automatic reference frame detection.

5 Installation

Installation ImageJ

ImageJ or Fiji — which is ImageJ with many useful plugins included — (recommended) can be downloaded from ImageJ NIH website or Fiji website, respectively. Download and installation instructions can be found on the aforementioned websites.

Installing MUSCLEMOTION macro

The macro can be installed from ImageJ/Fiji by using the Plugins>Macros>Install command and selecting the MUSCLEMOTION.ijm file. An icon with "MM" will now appear on the ImageJ/Fiji toolbar (see Figure 4).

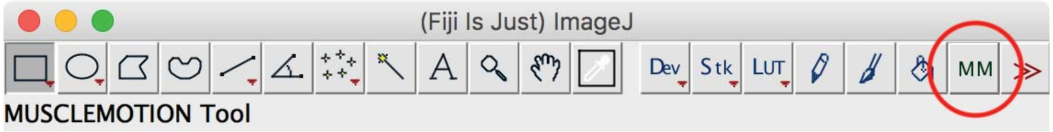

Figure 4: ImageJ/Fiji toolbar button for MUSCLEMOTION.

Increase the memory for ImageJ/Fiji

Since most image calculations require quite a bit of RAM, it is advised to increase the memory available for ImageJ/Fiji. To do so, select Edit>Options>Memory & Threads... and increase the maximum memory. Please take into account the maximal available RAM on the computer.

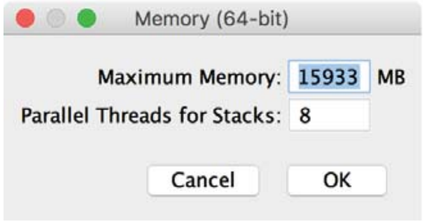

Figure 5: Memory increase.

Making a shortcut

To make sure ImageJ/Fiji shows the MUSCLEMOTION button after startup without the need to install it every time, copy MUSCLEMOTION.ijm to [imageJ/Fiji installation folder]/macros and rename it to "StartupMacros.ijm" (ImageJ) or "StartupMacros.fiji.ijm" (Fiji). The button should now appear each time ImageJ/Fiji is started. To run the macro simply click the MM button on the ImageJ/Fiji toolbar.

6 Running MUSCLEMOTION

This simple demo file allows the user to check whether everything is working properly and get to know the basic options of the algorithm. It is advised to run the demo before loading other recordings.

The demo files can be found in the folder demo. A demo file called demo_stack.tif is included and a subfolder called demo_results which contains the correct output files which should be compared after running MUSCLEMOTION with the default settings.

Wizard

Once MUSCLEMOTION has been started, a wizard window will guide the user through the customisation of the analysis settings. Default settings have been previously optimised for stem cell derived cardiomyocytes. If selected, advanced options will be shown in subsequent windows and will allow to customise parameters for multiple purposes.

A. Batch Analyses MUSCLEMOTION can perform analyses in batch throughout multiple directories (max. 2 directories deeper than the starting directory). If ”Yes” is selected, batch analyses will be performed on all the files contained in the chosen directory (including subdirectories).

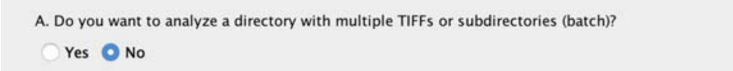

B. TIFF Images Sequence Some cameras only allows the recording of multiple individual images instead of a combined movie/stack. Select ”Yes” if the recording spans over multiple individual image files.

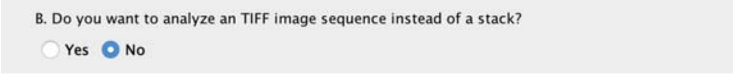

C. Gaussian Blur Some biological samples, as adult cardiomyocytes, have highly repetitive structures that can profoundly influence the linearity of MUSCLEMOTION. The application of a Gaussian blur cancels out the effects of repetitive patterns on the output precision. Select ”Yes” if you want to apply a Gaussian blur ($\sigma = 10$) to the movie. The effects of the added Gaussian blur will be visible in the image stack during the analyses.

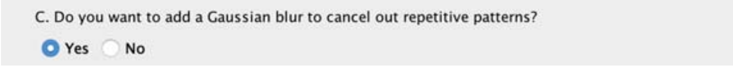

D. Frame Rate Here you have to insert the frame rate at which the recordings have been taken. This is important since the time scale of the output relies on this input parameter. Please note that batch analyses must be performed on movies recorded with identical frame rates.

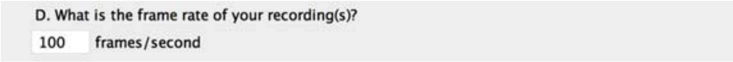

E. Speed Window The *Speed Window* defines the frameshift in calculating the measure of contraction speed (”running difference”). The higher the value, the smoother the curve, but it will also introduce a shift in time and amplitude. It is very useful with high frame rate recordings. We recommend a *Speed Window* of 2-5 frames every 100 fps to have a powerful but not intrusive filter in your outputs.

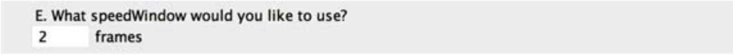

F. Noise Reduction Noise reduction is done by selecting the areas contributing most to the contraction transient. By default, this setting is active from the beginning of the recording to the end of the recording. If you prefer to avoid noise reduction, select ”No”. If you prefer to customise the interval (in frames) over which the noise reduction will take place, seek for the advanced options.

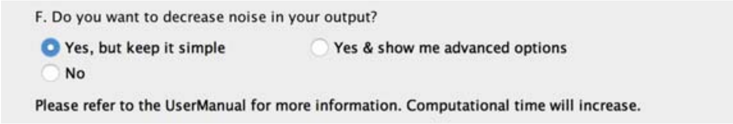

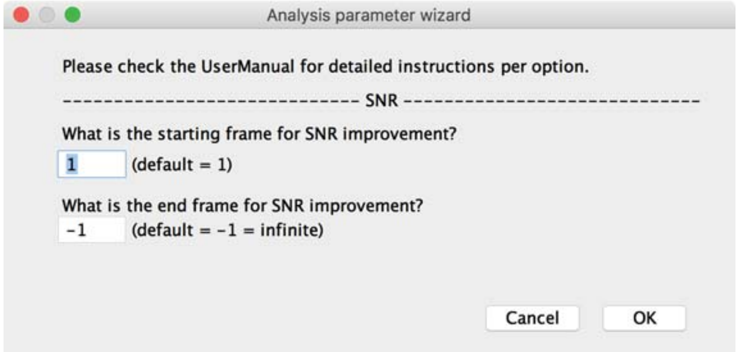

G. Automatic Reference Frame Detection MUSCLEMOTION can automatically detect the reference frame, and this option is active by default. If you do not want an

automatic reference frame selection, press ”No” to manually pick the reference frame in the movie with the built-in slider. The advanced options allow a customisation of the reference frame selection interval and thresholds. Specifically, the number of low points for automatic reference frame detection refers to the number of baseline values (frames) among which a certain number of point close to the unity line will be identified (respectively the blue and red dots in Figure S4 in the Paper).

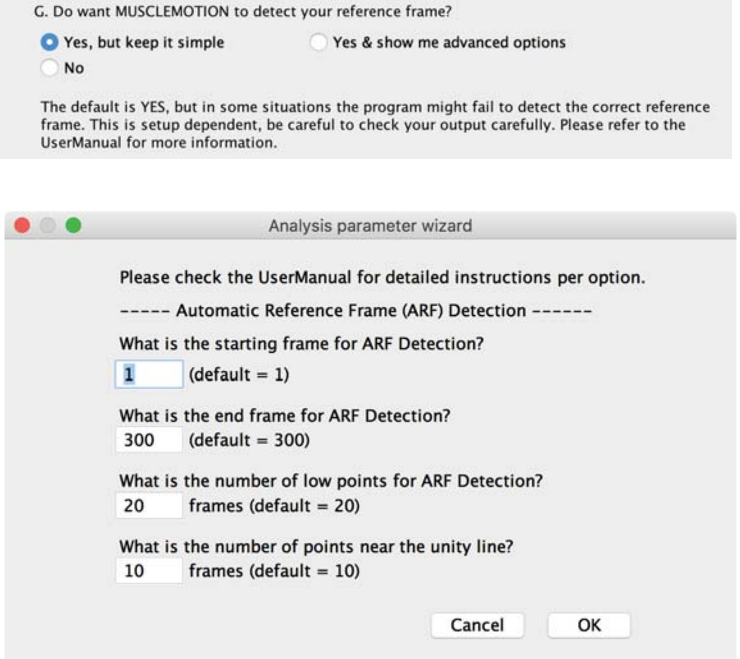

Figure 6: Advanced options of the Reference Frame.

H. Transient Analysis MUSCLEMOTION allows the automatic quantification of contraction profiles. Advanced options include the possibility to modify both the temporal and amplitude thresholds for the automatic identification of contractile peaks. Further options allows to select several contraction percentages as output as well as the fine-tuning of baseline and noise values. If the beating frequency of the sample is high (diastolic phase ¡¡ systolic phase), please select ”Yes” in the last option; this will automatically select the minimum baseline value before each peak.

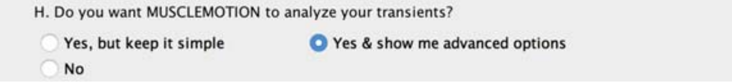

Proceed with the analysis by FIRST selecting the directory in which the

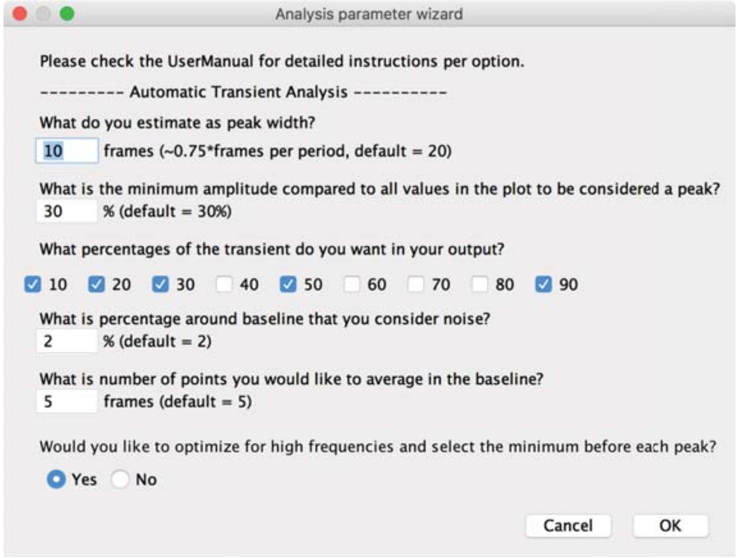

Figure 7: Advanced options of the Transient Analysis.

results will be saved and, AFTER THIS, the directory/file that have to be analysed.

7 Outputs

Once the analysis process has started, MUSCLEMOTION creates a directory in the selected folder and fills it with all the output files. A Log window allows to track all the steps in the analysis process and will be saved automatically at the end of the analysis.

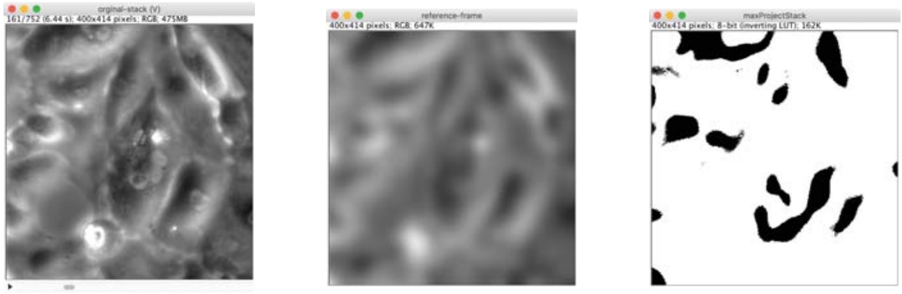

(a) (b) (c)

Figure 8: Original movie, reference frame and binary contracting areas.

Output Files

For each movie analysed, MUSCLEMOTION will generate 7 files, described in details below.

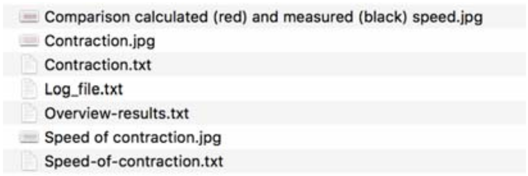

Comparison calculated (red) and measured (black) speed.jpg: This image overlays the calculated speed (red) and the measured speed (black) to have a rough linearity check on the output. A good overlap between the two traces indicates that MUSCLEMOTION worked within boundaries and, thus, the reliability of the results is granted. In case these two traces were misaligned, please update the settings by adding Gaussian blur or verify that the minimum requirements for frame rate and camera settings are fulfilled.

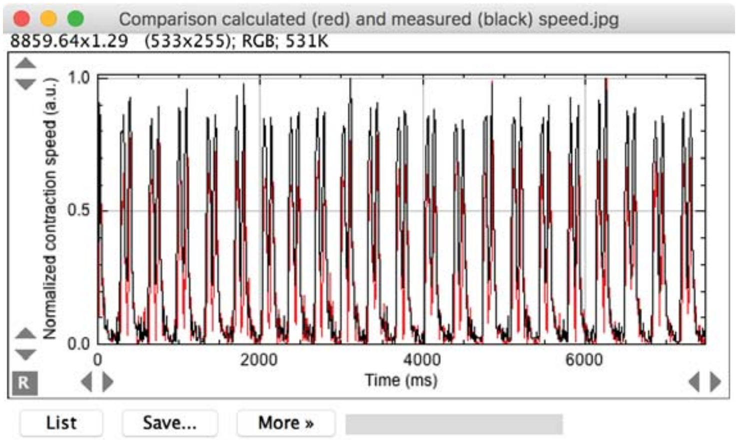

Contraction.jpg: This graph contains a low-resolution representation of the contraction profile. Vertical red lines indicate the temporal position of the identified contraction peak, while horizontal/oblique lines graphically connect the *percentages* of the transient previously chosen in the wizard procedure. Blue circles in diastole indicate the points identified and used as baseline to quantify the contraction parameters. In case the *high-frequency transient detection* option has been previously activated in the wizard, a blue short segment marks the selected baseline value.

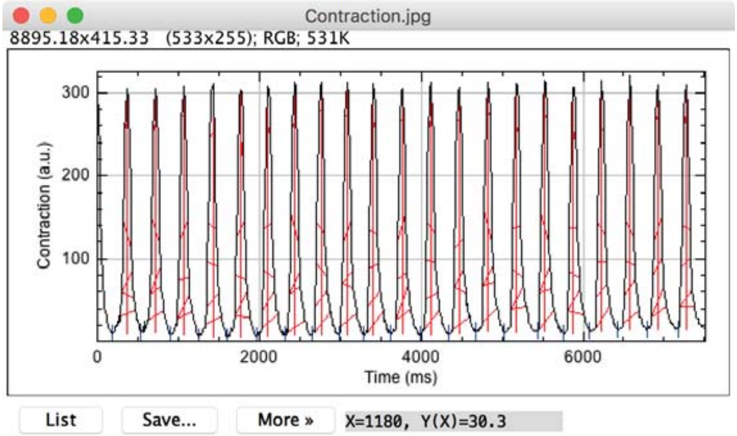

Contraction.txt: This file contains the time course profile of the contraction. This values can be imported in other software for the generation of high-resolution graphs.

Log_file.txt: This is the log file that contains a summary of the valuable information useful to identify and reproduce the analysis. These include time stamps and duration, a printout of the values of the used settings, the position in frames of the reference frame and of the peaks.

Overview-results.txt: This file is structured as a table with headers and contains the quantification of the parameters extracted from contraction profiles.

Speed of contraction.jpg: This graph contains a low-resolution representation of the speed of contraction profile.

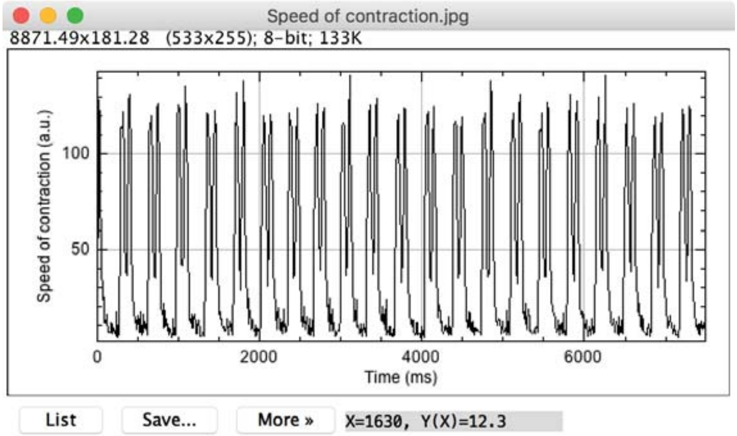

Speed-of-contraction.txt: This file contains the time course profile of the speed of contraction. This values can be imported in other software for the generation of high-resolution graphs.

8 Troubleshooting

Incorrect reference frame Sometimes MUSCLEMOTION fails to automatically detect the reference frame. If this happens, please adjust the parameters for the automatic selection in the advanced mode or manually select the reference frame.

9 How to cite

If you use MUSCLEMOTION in your publication, please cite it as: "**Sala L et al.**, Versatile open software to quantify cardiomyocyte and cardiac muscle contraction *in vitro* and *in vivo*, *bioRχiv*, 2017, doi: 10.1101/160754".

10 Contact

If you have a specific bug report for MUSCLEMOTION or would like to actively participate in developing please email the authors:

Berend J van Meer, MSc

Luca Sala, PhD
